# Composite material with enhanced recyclability as encapsulant for photovoltaic modules

**DOI:** 10.1016/j.heliyon.2023.e20048

**Published:** 2023-09-09

**Authors:** Francisco J. Cano, Gorka Imbuluzqueta, Naiara Yurrita, Jon Aizpurua, Juan M. Hernández, Werther Cambarau, Oihana Zubillaga

**Affiliations:** TECNALIA, Basque Research and Technology Alliance (BRTA), Mikeletegi Pasealekua 2, 20009, Donostia-San Sebastian, Spain

**Keywords:** Photovoltaic module, Encapsulant, Composite material, Recyclability

## Abstract

Encapsulation of photovoltaic cells was carried out using a transparent glass fiber reinforced composite with enhanced chemical recyclability based on a matrix of an epoxy resin containing cleavable functional groups. The current-voltage curves showed a decrease of 6.3% on the short-circuit current (Isc) after encapsulation of the cell, lower than the one observed for the reference non-recyclable standard epoxy composite. Its performance stability under thermal cycling, ultraviolet (UV), and damp-heat exposure was evaluated and compared with the one of the reference standard epoxy. Both resins showed good stability performance under UV exposure and thermal cycling accelerated aging. Moreover, a power loss below the 5% allowed by the photovoltaic standard was observed for the recyclable resin after 1000 h of damp-heat exposure, even the pronounced loss of 4.7% in power remains a concern. Regarding the recyclability, the composite was dissolved in acetic acid dissolution and glass fiber fabrics were successfully recovered. A new module was manufactured with these fabrics, showing this time a loss of 12% in Isc comparing with the non-encapsulated cell. Further work will consider improving the moisture barrier properties of the composite, and adjusting the recycling conditions to allow component recovery valid for new modules.

## 1 Introduction

Crystalline silicon-based photovoltaic (PV) modules consist of laminates of a multilayer polymer back sheet, a glass or polymer front sheet, and silicon cells encapsulated in an elastomer, commonly ethylene-vinyl acetate (EVA) [1,2]. With the increasing volume of these modules reaching the end of their operational life, their recycling has become an environmental and economic issue. Recycling of PV modules combines chemical, physical and thermal processes, and may allow the recovery of valuable materials. However, several technical, market and social challenges are to be solved in the implementation of large-scale module recycling [[3], [4], [5]]. Beyond recycling, an alternative approach for a circular economy in PV modules is to consider designing for extended product durability [6]. Additionally, new module designs and materials are being developed in order to promote their reuse and recycling at the end of their lifetime. Some of these last concepts include double encapsulation, cell interconnection via vacuum pressure without soldering and lamination, or substituting EVA with thermoplastic encapsulants [5].

Further, an emerging approach to enhance the recyclability in PV modules is the cell encapsulation in glass fiber reinforced epoxy composite materials instead of current glass laminates. Composite materials with acceptable optical transmittance [[7], [8], [9]] allow replacing not only the back sheet of the standard modules, but also the encapsulant and front sheet. Thus, lightweight and monolithic PV modules [[10], [11], [12], [13]] are enabled for their integration in urban areas covering building or mobility applications [14,15].

Recycling of fiber reinforced composite materials is being extensively studied in order to decrease landfilling and incineration, and boost recovery of the reinforcements and matrices [16,17]. Processes are being studied to mechanically, thermally and chemically recycling both thermoset and thermoplastic composites. Thermoplastic matrices show some advantages regarding recyclability, as reprocessing after mechanical crushing, or matrix and fiber recovery after dissolution process [17]. In the contrary, standard thermoset resins show a covalently cross-linked polymer network requiring more drastic conditions to be recycled. They can only be used as fillers after mechanical crushing, fiber damage has been observed after thermal treatment, and matrices are generally degraded and unable to be recovered when chemical treatments are implemented [17,18]. However, thermosets can be designed to be more easily degradable enabling their chemical recycling to obtain reinforcements, matrix related polymers and valuable components encapsulated in the composite [[18], [19], [20]]. The chemical recycling consists in solvolysis, which implies breaking the covalent bonds of the cured resin through chemical reaction with solvents [17]. Degradable thermosets are based on weak bonds, covering a wide chemical range of labile linkages as ester, sulfur containing bonds as disulfide, nitrogen containing structures, acetal linkages, and other many groups [19,[21], [22], [23]]. Between thermosets, epoxy matrices incorporating amine hardeners with acid-cleavable ether groups have been developed and tested. After treating the resulting composite in acetic acid solution, the carbon fibers were separated and a thermoplastic epoxy polymer was obtained after neutralization of the acidic solution [18,24].

Additionally, for its uptake as encapsulant in PV modules, beyond optical transmittance and recyclability at the end-of-life, the composite material should meet the durability requests of the modules [1,25]. During the operational lifetime, PV modules are exposed to environmental stresses as ultraviolet (UV) radiation, temperature variations, moisture, mechanical loads, soiling or diverse chemical pollutants. Delamination and discoloration are usual aging phenomena observed in field installed crystalline silicon modules encapsulated with standard system made of glass and EVA [[26], [27], [28]]. The same environmental factors are also degrading the composite materials [[29], [30], [31], [32]], potentially affecting the photovoltaic performance of PV modules when used as encapsulants. Particularly, accelerated aging of standard epoxy composite used as PV encapsulants were analyzed by the authors in previous works [[10], [11], [12]], observing acceptable power losses according to regulatory PV framework. However, the labile bonds incorporated in recyclable epoxy matrices may present sensitivity to hydrolysis [22], and thus, compromise the operational performance of the modules under humidity conditions.

The present work studies the encapsulation of crystalline silicon cells in glass fiber reinforced composite material with an epoxy matrix containing cleavable ether groups. The aim was to provide the encapsulating material and PV modules with enhanced chemical recyclability while retaining photovoltaic performance and durability.

## 2 Experimental

### 2.1 Materials and cell encapsulation

The modules were manufactured using monocrystalline silicon cells with a size of 156 mm × 156 mm, containing 5 busbars. Prior to encapsulation, soldering of the ribbons was performed using a tabber-stringer. The ribbons were further connected by hand soldering to obtain the positive and negative electrical terminals to be taken out from the module for characterization purposes. The manufactured photovoltaic modules consisted of one cell (monomodules), and were cut to a size of 200 mm × 200 mm.

The encapsulation of the photovoltaic cells was carried out using linear vacuum resin infusion process. As reinforcement, a glass fiber fabric with a 300 g/m2 (0/90°) areal weight was used. The reinforcement layout consisted of 3 layers placed at both, the front and back of the cell. As composite matrix, an epoxy resin system with amine base hardener and cleavable chemical groups in its composition was used. As a control reference, modules were manufactured with a standard resin system based on a clear bisphenol-A epoxy and an amine based crosslinker. The details of the manufacturing process were described previously by the authors [10].

12 monomodules were manufactured for accelerated aging tests covering thermal cycling, UV and damp-heat exposure. For each test, 3 monomodules were made with the resin with enhanced recyclability and 1 monomodule with the standard epoxy resin. Further, 2 additional monomodules, one with the cleavable epoxy and one with the standard epoxy, were manufactured to study the recycling performance.

### 2.2 Module characterisation

Electroluminescence (EL) images were obtained after cell encapsulation with the aim to identify potential cell or interconnection failures. The modules were forward biased through a current-voltage source prior image acquisition with a DSLR camera.

Current-voltage (IV) curves were measured for each monomodule using solar simulator (ABET-Sun3000). At least three measurements were carried out in each monomodule, using the average values for data analysis. The recorded IV parameters were short-circuit current (Isc), open-circuit voltage (Voc), power at maximum point (Pmp) and fill factor (FF). IV measurements carried out onto bare cells and corresponding monomodules allowed calculation of the electrical losses due to the composite encapsulation.

Further, the External Quantum Efficiency (EQE) of the cells and modules was measured (Bentham-PVE300). EQE values were obtained every 5 nm in the 300–1300 nm range. The illuminated spot had a size of 3.5 mm × 1 mm. Measurements were taken out at four points, in a line at the centre of the cell, in the four different spaces limited by busbars. The illuminated spot was located between the fingers, in cell areas free of metallization. The average value of these four points is plotted and used for the analysis of each cell and module. Using a template allowed placing the illuminated spot in the same position in cells and corresponding modules.

### 2.3 Accelerated aging tests

The monomodules were exposed to thermal cycling (TC), UV radiation and damp-heat (DH) accelerated aging conditions according to IEC-61215:2021 standard [25] to evaluate their performance stability.

The exposure to thermal cycling and damp-heat conditions was carried out in a climatic chamber (CTS C-70/350). The thermal cycling test covered 200 cycles between −40 °C and 85 °C. The damp-heat test comprised an exposure of 1000 h to 85 °C and 85% relative humidity. Concerning the UV exposure, it was performed in a Q-UV chamber (Q-Panel Company) at 60 °C for 380 h, achieving a final radiation of 15 KWh/m^2^.

The weathering evolution was monitored by EL, IV curves and EQE measurements as above described. Additionally, in the damp-heat test, the visual appearance was evaluated by colour measurements. CR-10 portable colour reader was used (Konica Minolta-d/8° sphere geometry, 10° standard observer, D65 standard illumination source, 8 mm spot size). The colour was measured on module surface, out of the cell and bus locations. b* chromatic coordinate values (blue-yellow axe) from CIE system were analyzed.

This characterization was carried out previous to exposure and after various aging periods.

### 2.4 Recyclability test

A first qualitative trial to assess the chemical recyclability of the resin under investigation was carried out. The aim of the recycling process to be developed was i) to isolate the cell and connections from the composite and ii) to recover the glass reinforcement.

The monomodule made of cleavable epoxy was dissolved in a solution of acetic acid at 50%, maintaining the module in the bath during 24 h at 50 °C. The module made of standard epoxy resin was exposed to the same solving conditions as reference sample for comparison purposes.

After recycling, the recovered glass reinforcement fabrics were used to manufacture a monomodule containing a back-contact cell, with the previous fiber lay-out and standard epoxy resin, following the same manufacturing process used for the pristine modules. This particular cell was selected because of its size (125 mm × 125 mm), smaller than the standard crystalline silicon cells used in the previous tests. This cell size allowed carrying out the infusion process with the fabrics of limited size recovered from the monomodule. The recovered fabrics presented the size of the original module, which was not large enough to allow an infusion process to manufacture a module of the same size. Additionally, the module, and the corresponding fabrics, were cut in the zone where electrical connections are retrieved from the module, to facilitate the recycling dissolution process. Thus, and because of its dimensions, encapsulating the same cell type used in pristine modules and which would be more representative, was not possible. IV curve measurements were carried out in both, the cell and the monomodule.

## 3 Results and discussion

### 3.1 Module manufacturing and characterisation

**Fig. 1:**
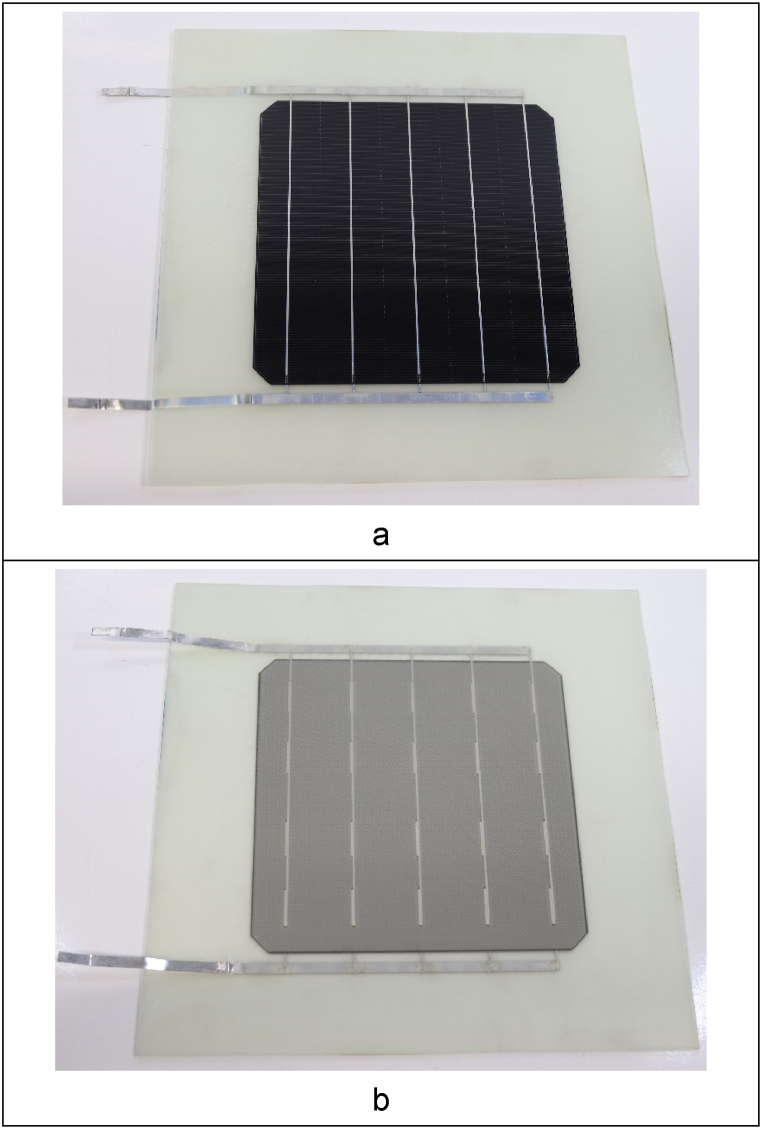
Picture of a cell encapsulated in composite with cleavable epoxy, front (a) and back (b) sides.

The manufactured monomodules showed a thickness in the range of 1.7 mm, being the cells, ribbons and bussing fully embedded in the composite. Fig. 1a shows the front side of the module made of cleavable resin, whereas its back side is depicted in Fig. 1b. The resin infusion proceeded smoothly in presence of cells, filling adequately the mould in all cases. No significant defects such as cell breakage or cell-composite delamination were detected during in the qualitative assessment of the monomodules. The appearance of the modules made of cleavable and standard epoxy was similar at naked eye.

**Fig. 2:**
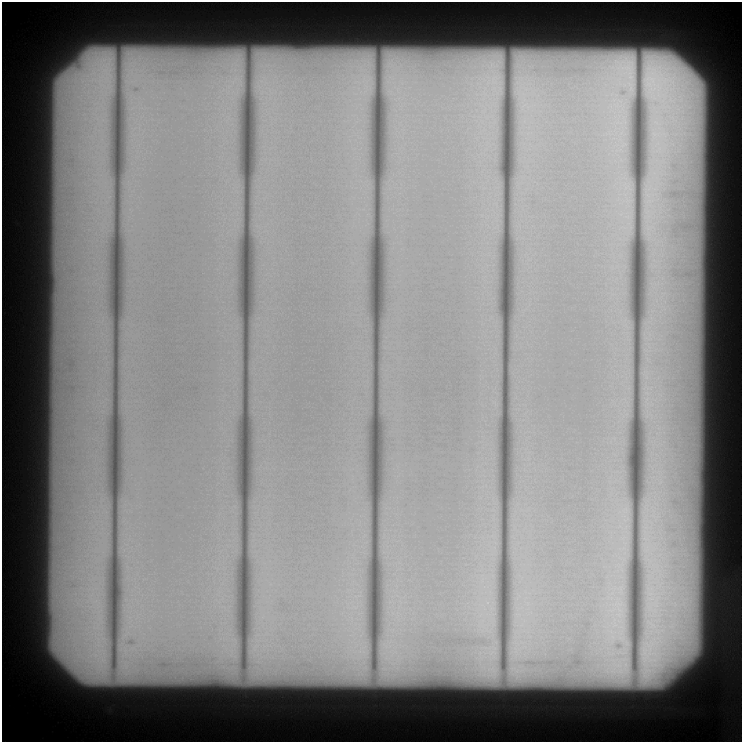
Electroluminescence image of one of the cells encapsulated in composite.

EL images confirmed that the cells did not present any significant damage due to the encapsulation process or contact with the resin systems. An EL picture of one of the monomodules can be observed in Fig. 2.

**Fig. 3:**
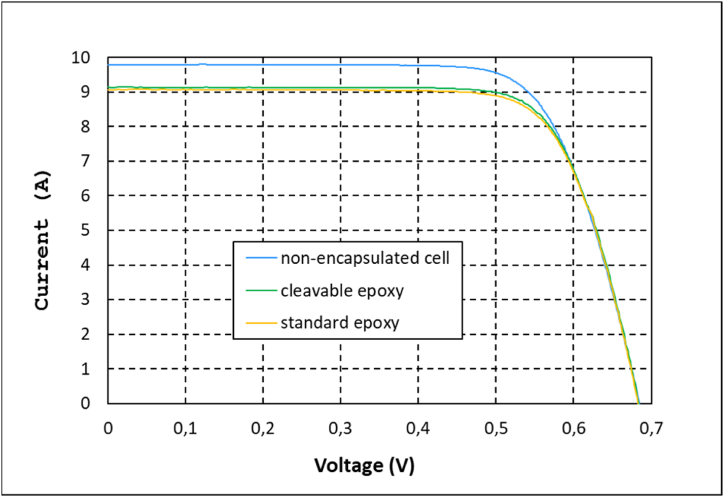
IV curves of bare cell and after encapsulation in recyclable and standard epoxy composite.

IV curves for the non-encapsulated bare cell and monomodules with recyclable and standard epoxy composites are depicted in Fig. 3. The corresponding values of the IV parameters are presented in Table 1. The data of the monomodules with the composite encapsulant based on the recyclable epoxy resin showed an electrical loss in short circuit current (Isc) of 6.3% (SD 0.20) when comparing the electrical performance before and after encapsulation. This value was slightly lower than the one obtained for the monomodules with standard epoxy composite, which presented a decrease of 7.2% (SD 0.17). Similar trend was observed when analysing the Pmp losses due to encapsulation.

**Table 1:** IV parameters of the bare cell and encapsulated in standard and recyclable composite.

IV paremeter	Isc (A)	Voc (V)	Pmp (W)	FF (%)
Bare cell^a^	9.78	0.69	4.87	72.41
Cleavable	9.16	0.68	4.65	75.23
Standard	9.07	0.68	4.61	74.35

a With connected ribbons and buses.

**Fig. 4:**
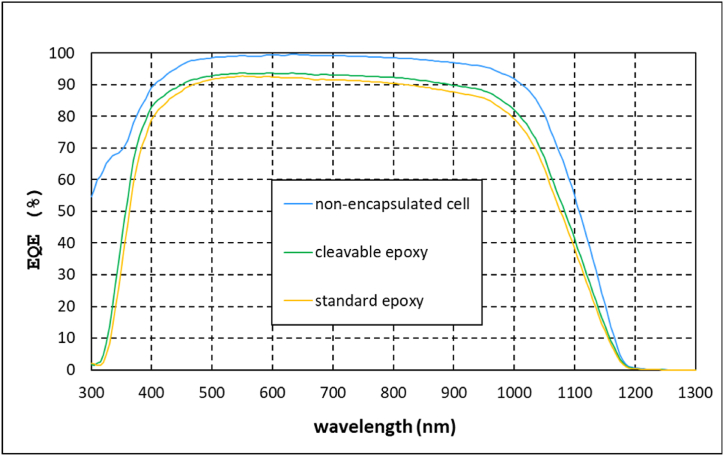
EQE spectra of the bare cell and the monomodules as a function of the encapsulant resin.

The same trend was observed in the EQE spectra (Fig. 4), showing the modules with recyclable epoxy a slightly higher EQE response along the whole wavelength range of the spectrum. This better performance of the recyclable composite in EQE and Isc response could be attributed to a better optical compatibility between the cleavable epoxy resin and the glass reinforcement, in terms that the value of the refractive index of this epoxy would be closer to the one of the used glass reinforcement. In any case, this should be verified by transmittance and reflectance measurements.

The obtained results suggested that the recyclable epoxy would be the preferred option in front of the standard one, offering the highest electrical performance. However, the performance stability of the recyclable resin should be as well considered, as shown below.

### 3.2 Accelerated aging test results

**Fig. 5:**
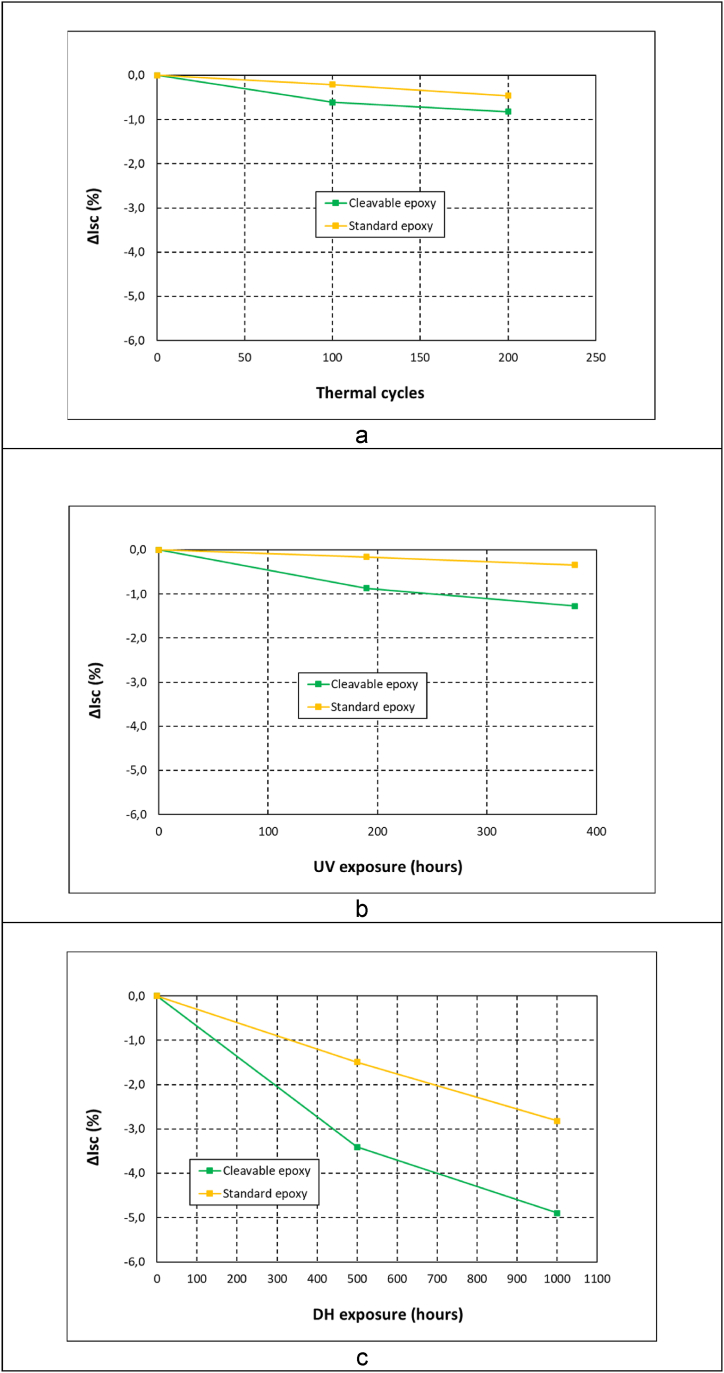
Short-circuit current loss evolution for different resins in (a) thermal cycling, (b) ultraviolet and (c) damp-heat exposure.

**Fig. 6:**
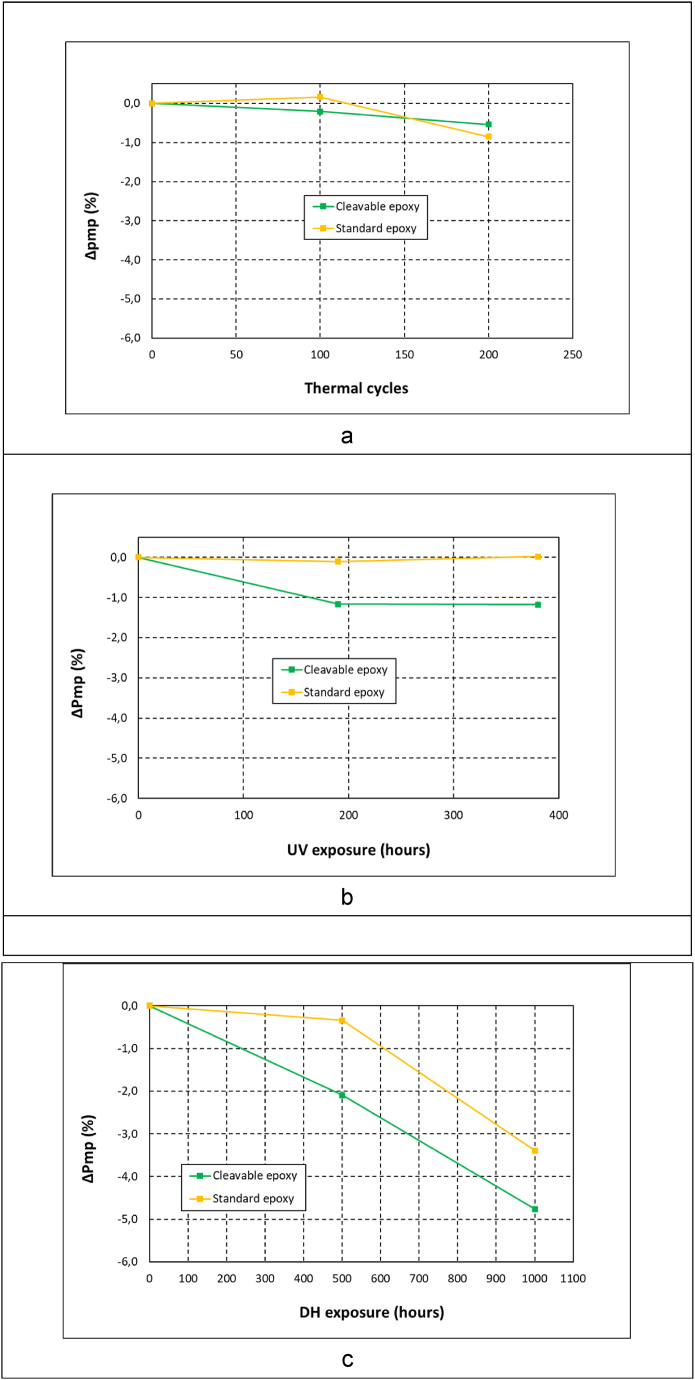
Power loss evolution for different resins in (a) thermal cycling, (b) ultraviolet and (c) damp-heat exposure.

The Isc (Fig. 5) and Pmp (Fig. 6) parameters from the IV curves allowed analysing how the module electrical performance was changing during aging exposure. The observed variation under thermal cycling, UV and damp-heat exposure for both resins is presented in Fig. 5, Fig. 6. Overall, the composite with recyclable epoxy with cleavable groups showed a worse stability than the standard epoxy in the three testing conditions. Further, the worst aging performance was observed in damp-heat tests for both resins.

For the thermal cycling test, both resins presented a loss below 1% in Isc (Fig. 5a) and Pmp (Fig. 6a). The Isc gradually decreased in the recyclable modules, reaching a final value of 0.8%, whereas the standard epoxy showed a loss of 0.5%. These observed changes can be considered not to be significant, as they are below the measurement accuracy of the technique, around 1%. Similar case was found when analysing the UV radiation exposure, with electrical losses in Isc (Fig. 5b) and Pmp (Fig. 6b) close to 1%. In any case, the results suggested a slightly worse performance of the recyclable epoxy with a loss in Isc of 1.3% at the end of the test. These values are in the range of the aging observed by the authors for UV and thermal cycling exposure in previous works [10,12].

The aging in the damp-heat test was more marked for both resins. For the recyclable module, a sharp current decrease already occurred after 500 h of exposure in Isc (Fig. 5c) and Pmp (Fig. 6c). A loss of 3.4% was observed in Isc, more than twice than the modules made of standard epoxy composite, which showed a reduction of 1.5%. Similar evolution was observed in the Pmp, being the decrease for the recyclable epoxy of 2.1%, whereas the change for the standard epoxy was below the measurement accuracy. After 1000 h exposure, the observed Isc decrease was even more pronounced, being significantly higher for the cleavable epoxy matrix. A final loss of 4.9% was measured for recyclable resin, whereas the standard epoxy showed a lower value of 2.8%. Regarding Pmp values, the loss reached a value of 4.7% and 3.4% for the recyclable and standard composite respectively. This significant electrical degradation observed for the modules made of cleavable epoxy is a concern in order to fulfil the IEC regulation [25], which allows a power loss up to 5% in photovoltaic modules. Further investigation is required to analyse this worse performance of the resin with cleavable functional groups, and be able to understand how the high temperature and humid conditions affects the degradation of this resin, affecting its optical properties, permeability and other essential features to work as photovoltaic encapsulant.

**Fig. 7:**
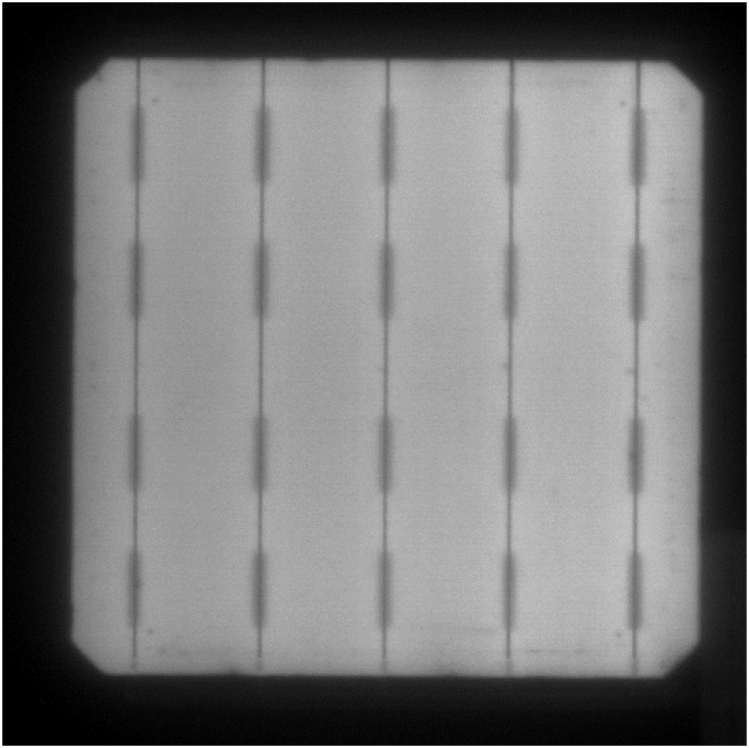
Electroluminescence image of a monomodule encapsulated in recyclable resin after 1000 h damp-heat exposure.

This degradation was further studied by EL analysis of the modules. The EL image of the monomodules with cleavable epoxy matrix did not show any significant damage in the cell. The picture of the same cell from Fig. 2 after 1000 h damp-heat aging is presented in Fig. 7, not observing major differences between the two images.

**Fig. 8:**
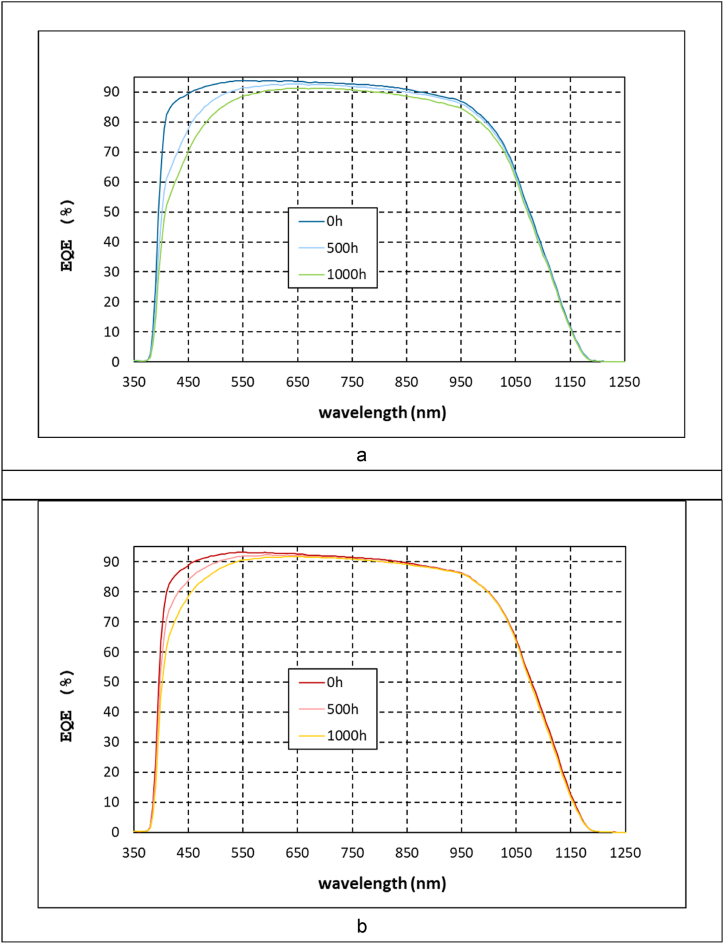
Evolution of EQE spectra of the modules during damp-heat exposure for (a) cleavable resin and (b) standard resin.

**Fig. 9:**
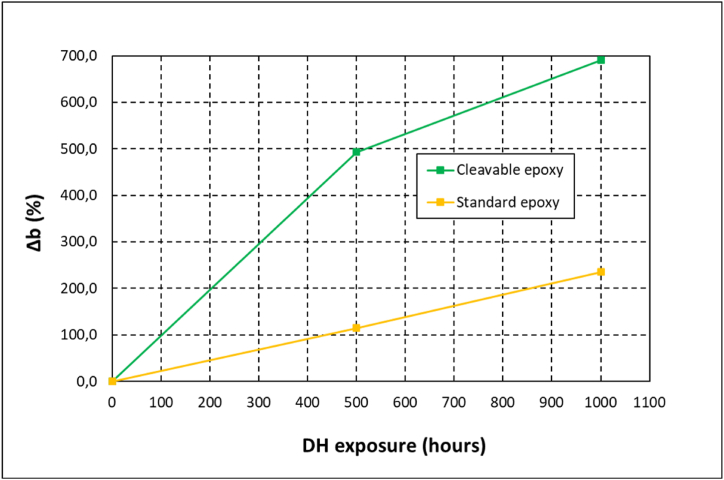
Variation of colour component b* of the modules during damp-heat test for the cleavable and standard resin composites.

The study to analyse the origin of the electrical loss continued with EQE characterization of the aged modules. EQE spectra were acquired at the same exposure intervals when the IV curves were measured. Fig. 8 plots the spectra for both resins after 500 h and 1000 h of damp-heat test. The EQE results were in accordance with the Isc losses, observing a more pronounced degradation in the modules with the cleavable epoxy. Focusing on the standard epoxy, the degradation process is located in wavelength values below 600 nm, meaning that possibly chromophoric compounds that absorbs in the 300–600 nm spectral region are formed in the resin during damp-heat exposure. This optical degradation of the composite would not penalize the light reaching the cell in higher wavelengths, being the power loss moderate. In contrast, the modules with cleavable epoxy showed an overall and more pronounced decrease in EQE spectra along the whole measured wavelength range. This could indicate that in this case, the degradation is more complex, involving probably various phenomena such as the formation of chromophoric groups, other resin deterioration mechanism and even cell damage. Additionally, the evolution of the b* chromatic component was monitored (Fig. 9), observing that the yellowing suffered by the composite with cleavable epoxy was up to three times higher, which also may indicate the higher optical degradation presented by the recyclable composite.

### 3.3 Recycling performance

**Fig. 10:**
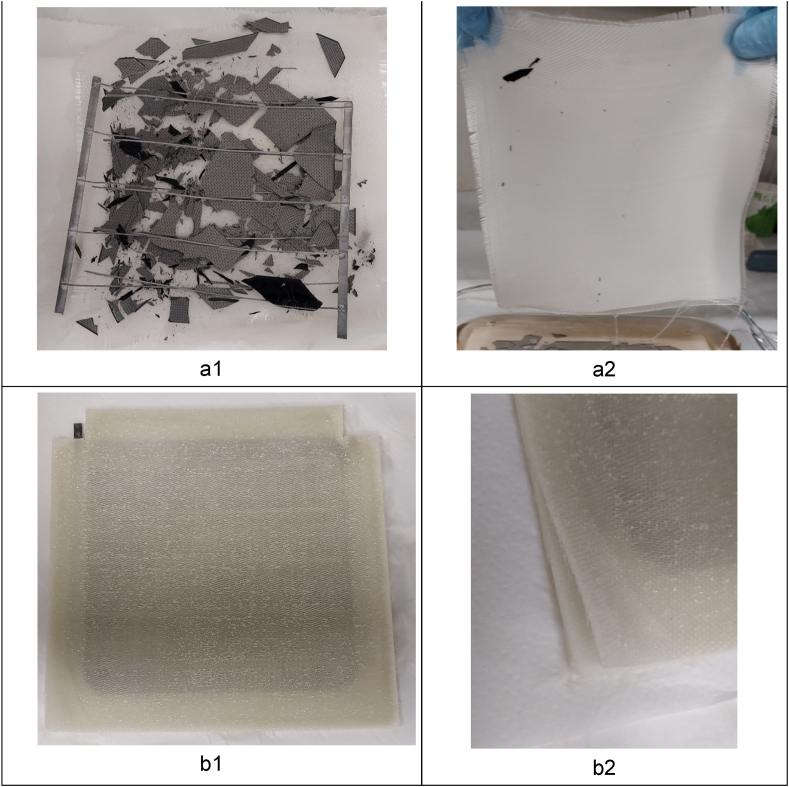
Composite monomodules after immersion in acetic acid solution: (a) monomodule made of cleavable resin separated in broken cell and connectors (a1) and reinforcement fabric (a2), and (b) monomodule made of standard resin with no separation of fabric layer (b1) (detail in (b2)) and still embedded cell.

The three components, i.e. reinforcement, matrix and cell, present in the monomodule comprising the cleavable epoxy were separated with no difficulty after its exposure to acetic acid solution (Fig. 10a). In contrast, in the monomodule made of standard epoxy, a slight wetting of the composite by the solution was observed, but no component was able to be separated (Fig. 10b).

Coming back to the cleavable epoxy composite monomodule, each of the six reinforcement fabrics was recovered and rinsed in distilled water. The cured resin was completely solved in the acidic solution. No further step to recover any polymer through precipitation was carried out. Regarding the PV cell, its surface was completely damaged, the wafer was broken into many pieces, and ribbons and buses were detached from the busbars.

Related to the recycling performance, the composite encapsulation offers the advantage of exposing the whole surface area to the chemical recycling solution, instead of solution access limited to the module edges that is present in glass-based PV panels. Additionally, the cell and connection components are separated from the resin, which may be advantageous with respect to EVA based modules, when recycling high-value elements as silver [4].

Future work remains on optimising solution composition, and process temperature and time. The influence of the number of reinforcement layers in recycling performance was also a parameter to be further studied. Additionally, the recycling performance of the modules after accelerated aging or at the end of the working life remains to be studied, when degradation is expected to occur, affecting the chemical structure of the resin, composite, or overall module, and possibly the recyclability features.

**Fig. 11:**
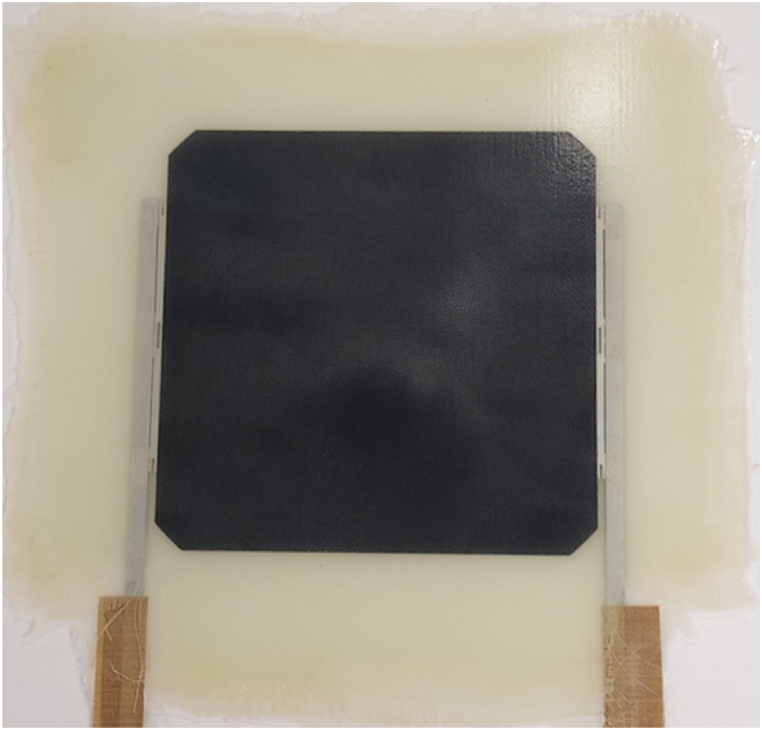
Back-contact cell encapsulated with recovered reinforcement fabrics.

A monomodule consisting of a back cell was manufactured with the recovered reinforcement fabrics (Fig. 11). During the infusion process, the resin flowed smoothly until complete mould filling. No cell breakage or delaminations were observed at naked eye during qualitative inspection. However, the module showed an uneven surface regarding optical appearance, with some slightly whitish regions distributed along the whole cell area. This phenomenon could be attributed to a non-homogeneous composition of the reinforcement, due to presence of i) non completely eliminated resin during recycling or fabric rinsing, or ii) some damage in fiber sizing during recycling dissolution process.

The short-circuit current (Isc) value of the module according to the measured IV curve was of 5.7 A. This means a decrease of 12% in Isc related to the non-encapsulated cell. These loss values were slightly higher than the ones of 8% observed by the authors in previous works for this kind of cells and encapsulant [12]. This higher electrical loss due to encapsulation is in accordance with the non-fully transparent composite surface observed in the module.

Further work comprises analysis and improvement of surface homogeneity, and studying the aging performance of the modules made of composite having the recovered fibers, with, between others, a possibly different resin-fiber interface.

## 4 Conclusions

A composite material with enhanced chemical recyclability made of glass-fiber and an epoxy resin containing cleavable functional groups was analyzed for its use as encapsulant of photovoltaic cells. Comparing with the baseline composite made of standard epoxy, the initial electrical performance of the new composite showed a lower Isc loss, with a value of 6.3%. However, its performance stability was worse than the one of the standard epoxy under the accelerated aging tests of thermal cycling, UV exposure and damp-heat. Particularly, it showed a pronounced loss of 4.9% in Isc after damp-heat test.

Concerning the recyclability, the composite was successfully dissolved in acetic acid dissolution and glass-fiber fabrics were recovered. The new module made of these fabrics and standard epoxy showed a non-homogeneous surface and a loss of 12% in Isc comparing with the non-encapsulated cell.

Considering these results, future work will be focused on improving the damp-heat stability of the composite, in a trade-off with a successful recyclability.

## Author contribution statement

Francisco J. Cano: Conceived and designed the experiments; Performed the experiments; Analyzed and interpreted the data; Wrote the paper.

Gorka Imbuluzqueta, Naiara Yurrita: Performed the experiments; Analyzed and interpreted the data; Contributed reagents, materials, analysis tools or data.

Jon Aizpurua, Juan M. Hernández: Performed the experiments.

Werther Cambarau: Analyzed and interpreted the data; Contributed reagents, materials, analysis tools or data.

Oihana Zubillaga: Conceived and designed the experiments; Analyzed and interpreted the data; Wrote the paper.

## Data availability statement

Data will be made available on request.

## Declaration of competing interest

The authors declare that they have no known competing financial interests or personal relationships that could have appeared to influence the work reported in this paper.
